# The dynamics of disease mediated invasions by hosts with immune reproductive tradeoff

**DOI:** 10.1038/s41598-022-07962-2

**Published:** 2022-03-08

**Authors:** Matthew J. Young, Nina H. Fefferman

**Affiliations:** 1 National Institute for Mathematical and Biological Synthesis (NIMBioS), University of Tennessee, Knoxville, TN USA; 2 Department of Ecology and Evolutionary Biology, University of Tennessee, Knoxville, TN USA

**Keywords:** Biological models, Animal disease models, Ecological epidemiology

## Abstract

The modern world involves both increasingly frequent introduction of novel invasive animals into new habitat ranges and novel epidemic-causing pathogens into new host populations. Both of these phenomena have been well studied. Less well explored, however, is how the success of species invasions may themselves be affected by the pathogens they bring with them. In this paper, we construct a simple, modified Susceptible-Infected-Recovered model for a vector-borne pathogen affecting two annually reproducing hosts. We consider an invasion scenario in which a susceptible native host species is invaded by a disease-resistant species carrying a vector-borne infection. We assume the presence of abundant, but previously disease-free, competent vectors. We find that the success of invasion is critically sensitive to the infectivity of the pathogen. The more the pathogen is able to spread, the more fit the invasive host is in competition with the more vulnerable native species; the pathogen acts as a ‘wingman pathogen,’ enhancing the probability of invader establishment. While not surprising, we provide a quantitative predictive framework for the long-term outcomes from these important coupled dynamics in a world in which compound invasions of hosts and pathogens are increasingly likely.

## Introduction

Biological invasions play a critical role in shaping the ongoing community dynamics of established ecosystems^1,2^. While invasions are a natural process of population growth, they are also facilitated by increasingly common influences of land use change, climate change, and anthropogenic transport (whether purposeful or accidental) of novel species to non-native habitats^3–6^. The ability of native populations to repel invasion attempts, or to survive successful invasions, depends on a multitude of factors^7^ and are, of course, actively influenced by conservation/restoration efforts^8^.

Invasions can be even more complicated when the invasive species is itself a pathogen that can use members of native species as hosts. Recent work has explored the fascinating dynamics of parasitic invasions^9,10^. While clearly a special case of a well-studied scenario in which a novel predator (the infectious agent, whether parasite or pathogen) arrives to potentially decimate a native prey (the host), this has been much more thoroughly explored within the scope of epidemiological dynamics of the introduction of novel pathogens, rather than through the lens of invasion ecology. As a result, most of the focus has been on establishing criteria for whether a novel pathogen will either force the native hosts extinct (and thus likely die out in the new environment itself), establish itself in the native host population as a new endemic disease, or sweep through the native population in one or multiple outbreaks only to result in herd immunity that eradicates the disease from a remaining host population (which could then experience another outbreak after sufficient migration or the birth of new hosts)^11–14^. Some work has gone so far as to explore the evolutionary implications. Research has suggested that introduction of a pathogen into a novel host should select for the evolution of changes in virulence over time^15^. When endemic competition is high, selection should favor increased virulence, which however also increases the probability of either host extinction or self-limiting outbreaks^16–18^. On the side of the host, introduction of novel pathogens has been proposed as a sufficient sudden selective pressure that it could lead to evolutionary rescue effects^19–21^. While it is unusual to discuss host evolution without also considering pathogen evolution due to the relative mutability and generation times of most host/pathogen pairs, there has now been empirical evidence of such dynamics in wildlife populations; e.g.,^22^.

Returning to an ecological perspective, however, highlights a thus far less well studied aspect of the likely scenarios around the introduction of novel pathogens: they are likely to arrive as passengers within hosts who are themselves potential invaders, and subsequently infect the native hosts as well. Although some excellent papers have explored this direction^23–25^, more research is required to elucidate the complex interactions in these scenarios. The involvement of these infectious passengers vastly complicates the potential dynamics of invasion for the invading host, the carried pathogen, and the native host. Under traditional scenarios of the invasion of a potential competitor into an ecosystem, there are three possibilities: Invaders fail to establish and die out; Invaders establish and successfully invade, displacing the natives and forcing their population extinct; and Invaders successfully establish a population that then stably co-exists with the natives, potentially altering carrying capacity, but not truly threatening the persistence of the native population nor under ongoing risk of extinction due to founder/small population effects^2,26^. These same logical options exist for invasive hosts carrying novel pathogens that can also infect native populations, but each case then also branches into multiple subcases. In the case where the invading hosts fail to establish, the pathogen may die out with them (Note, their lack of success may be partially due to poor initial health due to harboring an active infection.), or may survive and become endemic in the native hosts. In the case where the invading hosts drive the natives to extinction the pathogen is likely to survive, but may go extinct in the new environment. In the case where the two hosts coexist, the pathogen may sweep through both native and invasive populations and then die out, it may become endemic in one population with occasional spill-over outbreaks into the other, it could alternate between outbreaks in each population, or it could become fully endemic in both populations. Real-world examples of these sorts of dynamics have already been described in several cases, such as in squirrels^27,28^, birds^29^, moose and deer^30^, and crayfish^31^.

Although most of the examples of disease-mediated invasions that have been considered in the literature are of direct-transmission diseases, vector-borne diseases are a strong candidate for such interactions due to the ability of vectors to enable interspecies transfer of disease^32^. Further, vectors provide a mechanism by which species can help spread a disease without being directly susceptible to it. For instance, mountain hares and red grouse do not directly compete over resources, but both are preyed upon by ticks carrying the flavivirus that causes louping-ill, which causes encephalitis in infected hosts. Although the hares have low susceptibility to louping-ill and do not spread the pathogen to the ticks, by providing blood-meals they support the growth of the vector population in the ecosystem, which then increases the rate of disease transmission among the vulnerable grouse^33^.

In any of these scenarios of host-pathogen co-invasion (pathogen failure, pathogen sweep, endemicity in one or both hosts), both the initial transient dynamics after host invasion and the ultimate stable outcomes for the host populations can be meaningfully impacted by the additional complexity of the pathogen. The last case, in which the invasive hosts manage to displace the native hosts, is the most complicated and also the most intriguing. In this scenario, the pathogen may play a critical role in decreasing the relative viability of the native hosts, who may have fewer co-evolved defenses against the pathogen than their current invasive hosts do. This is akin to predator release^29,34,35^, in which invasive populations actually experience better population growth in novel environments because of the absence of co-adapted predators, but in this case, it is to the detriment of the native population to not have such co-adapted protections against predators. Although this phenomenon has been described in parasites as apparent competition, apparent competition alone does not describe the complete set of scenarios possible because no observable trade-offs in population sizes or growth rates are needed. Rather than experiencing release, these populations are essentially deploying accidental biowarfare on the native populations they are invading^23^. We propose that these “wingman pathogens” (sometimes called “disease mediated invasions”^23^) may play a critical role in invasion dynamics and here present a model to study the case in which co-evolved invasive hosts carry a novel vector-borne pathogen with them into the habitat of a more disease-sensitive native population.

## The model

Following the work in^36^, we construct an epidemiological model which tracks the disease dynamics and population of two species of hosts following the introduction of a pathogen. The native host (hereafter simply referred to as “type 1”) is vulnerable to the disease, but due to being well adapted to the native habitat has high fecundity when uninfected. The invasive host (hereafter referred to as “type 2”), has coevolved defenses to the pathogen that increase both its tolerance of and resistance to the disease, but is not inherently as well-adapted to the habitat in the absence of infection (i.e., its intrinsic rate of growth in the new habitat is lower than that of the native).

Our initial conditions correspond to a population of uninfected type 1 hosts with a small number of both uninfected and infected type 2 hosts, representing an invasion by a novel competitor carrying a novel pathogen into the type 1 population. We consider a vector-borne pathogen, and make the simplifying assumption that there is an already abundant competent vector species in the habitat. (For this initial formulation, we considered a scenario of mosquito-borne infections in birds, such as avian malaria^37^ or West Nile virus^38^, to motivate concrete choices.)

The model couples two biological dynamics: the daily vector-borne spread of the disease among hosts, and a yearly host breeding cycle. We simulate in discrete time-steps that represent days using an SIR model taking into account the interactions between the disease, the two species of host, and the vectors. The model also includes a passive death rate for hosts of vectors, which increases for hosts while infected. While the vectors are assumed to breed daily, the hosts reproduce as part of an assumed annual breeding season, every $t_c$ time-steps (typically equal to 365). These dynamics were informed by considering an annually breeding bird population in a tropical environment, however, they are not meant to reflect the realism of any one biological system. They are chosen here merely to allow a clean interpretation of modeled scenarios. Future models should explore the impact of greater variety in the dynamics of possible vector and host reproductive patterns.

### Epidemiological model

The model tracks eight variables corresponding to combinations of host species and vectors with their infection status. Hosts may be of type 1 or 2, and are either susceptible to the disease ($S_1, S_2$), currently infected ($I_1, I_2$), or recovered ($R_1, R_2$). We assume that recovery is complete and recovered individuals suffer no residual effects from their infection aside from a lifelong immunity to becoming reinfected. (We later set the recovery rate for host type 1 to 0, so $R_1 = 0$ at all times, but leave it defined for the sake of generality.) For simplicity, we model using only one stage of infection in which individuals are both infectious and symptomatic. The model also tracks the status of the vector population, which may either be susceptible ($S_v$) or infected ($I_v$). We assume that vectors do not recover from the disease, but also suffer no negative effects from being infected, acting only as carriers.

For convenience of notation, we denote the total number of hosts

$$\begin{aligned} H = S_1 + I_1 + R_1 + S_2 + I_2 + R_2 \end{aligned}$$

and the relative frequencies of infection within their respective population

$$\begin{aligned} F_1 = \frac{I_1}{H}, F_2 = \frac{I_2}{H},F_v = \frac{I_v}{S_v+I_v} \end{aligned}$$

which allows some equations to be written more compactly. Table 1 shows a summary of these variables.

**Table 1:** Variables.

Variable	Description
$S_1,I_1,R_1$	Susceptible/Infected/Recovered host 1
$S_2, I_2, R_2$	Susceptible/Infected/Recovered host 2
$S_v, I_v$	Susceptible/Infected vectors
*H*	Total hosts
$F_1, F_2, F_v$,	frequency of infection for host 1/host 2/vector

The model also has several constant parameters that affect the dynamics. $\beta _j$ determines the probability that hosts of type *j* become infected when bitten by a single infected vector. We typically set $\beta _1 > \beta _2$, making type 2 hosts less likely to become infected.

Likewise, $\delta _j$ determines the probability that a vector becomes infected when biting an infected host of type *j*.

$b_j$ determines the bite rate for vectors on host type *j*. We assume that each vector bites the same number of hosts per day, so each vector’s probability of becoming infected depends only on the frequency of infection among hosts, while each host will be bitten more if there are more vectors.

$\gamma _j$ determines the proportion of infected hosts of type *j* that recover from the disease each day. We typically set $\gamma _1 = 0 < \gamma _2$, meaning infected hosts of type 1 do not recover, while infected type 2 recover after an average of $1/\gamma _2$ days.

$\mu _{j-}$ determines the daily death rate for uninfected hosts of type *j* and $\mu _{j+}$ determines the death rate for infected host of type *j*. We typically set $\mu _{1-} = \mu _{2-}< \mu _{2+} < \mu _{1+}$, meaning uninfected hosts have the same death rate regardless of type, infected type 2 have a higher death rate than uninfected hosts, and infected type 1 have the highest. (Both susceptible and recovered hosts are considered to be uninfected.) Table 2 shows a summary of parameters related to the SIR dynamics.

Equation 1 shows continuous ordinary differential equations approximating the dynamics. Note that the actual model instantiates these in discrete time-steps using the forward Euler method with $h = 1$.

1 $$ \begin{aligned}&\frac{dS_1}{dt} = - S_1 \beta _1 b_1 I_v /H - S_1 \mu _{1-} \\&\frac{dI_1}{dt} = S_1 \beta _1 b_1 I_v /H - \gamma _1 I_1 - I_1 \mu _{1+} \\&\frac{dR_1}{dt} = I_1 \gamma _1 - R_1 \mu _{1-} \\&\frac{dS_2}{dt} = -S_2 \beta _2 b_2 I_v /H - S_2 \mu _{2-} \\&\frac{dI_2}{dt} = S_2 \beta _2 b_2 I_v /H - I_2 \gamma _2 - I_2 \mu _{2+} \\&\frac{dR_2}{dt} = I_2 \gamma _2 - R_2 \mu _{2-}\\&\frac{dS_v}{dt} = \alpha _v H -S_v \delta _1 b_1 F_1 -S_v \delta _2 b_2 F_2 -S_v \mu _v\\&\frac{dI_v}{dt} = S_v \delta _1 b_1 F_1 + S_v \delta _2 b_2 F_2 - I_v \mu _v\\ \end{aligned} $$

**Table 2:** Parameters for SIR dynamics.

Variable	Description
$\beta _j$	Probability of infection when host type *j* is bitten by an infected vector
$\delta _j$	Probability a vector is infected when biting an infected host of type *j*.
$b_j$	Bite rate on host type *j* (number of times bitten per day per mosquito divided among the host population)
$\gamma _j$	Recovery rate for host type *j*
$\mu _{j-}, \mu _{j+}$	Death rate for uninfected/infected hosts of type *j*
$\alpha _v, \mu _v$	Birth and death rates for vectors

Following a standard SIR model, susceptible hosts can become infected, and infected hosts become recovered, but each equation also contains a negative term corresponding to deaths. Thus, the total population of hosts is strictly decreasing in this time-frame. We assume that the vectors breed on a much shorter timescale than hosts, so we include a term for their births here, while host births are implemented by a yearly breeding event. We assume no vertical disease transmission, so all new vectors begin in the susceptible category. We assume that the daily birthrate for each vector increases with access to hosts, and decreases with competition among other vectors for hosts and breeding sites, so we set it equal to $\frac{\alpha _v H}{S_v + I_v}$, where $\alpha _v$ is a constant scaling factor. Since the birthrate for each vector contains the total number of vectors in its denominator, the total number of vector births in the population will simply be $\alpha _v H$.

A population with a larger number of hosts will be able to sustain a larger number of vectors. For a population with a constant number of hosts, the equilibrium vector population will be proportional to the number hosts: *aH* where $a = \frac{\alpha _v}{\mu _v}$ is the equilibrium vector density (number of vectors per host). For instance if $a = 2$, then in equilibrium there will be twice as many vectors as hosts. Given a fixed number of hosts, the population of vectors will asymptotically approach the equilibrium value. In practice the total number of hosts is constantly changing, so the population of vectors will chase after this moving equilibrium, though for our standard parameters $\alpha _v$ and $\mu _v$ are sufficiently large such that this will occur on a short timescale, and the population of vectors remains close to the current equilibrium value.

### Breeding event

Table 3 shows a summary of parameters related to the breeding event. Every $t_c$ days (typically 365), a breeding event occurs according to the following process.

**Table 3:** Parameters for breeding event.

Variable	Description
$\alpha _{j-}, \alpha _{j+},$	Birth rate for hosts when uninfected/infected
$\kappa$	Carrying capacity
$t_c$	Number of days between each breeding cycle

Let

$$\begin{aligned}&\Delta S_1 = t_c \alpha _{1-}(S_1+R_1)+t_c\alpha _{1+} I_1 \\&\Delta S_2 = t_c \alpha _{2-}(S_2+R_2)+t_c\alpha _{2+} I_2 \\ \end{aligned}$$

be the number of new host offspring of each type born this generation. In order to maintain consistency of temporal units among the parameters, each birthrate parameter is multiplied by $t_c$. Let *H* be the current total number of hosts. Let

$$\begin{aligned} c = {\left\{ \begin{array}{ll} 0 &{} \hbox {if } H \ge \kappa \\ 1 &{} \hbox {if } H + \Delta S_1 + \Delta S_2 \le \kappa \\ \frac{\kappa -H}{\Delta S_1 + \Delta S_2} &{} \hbox {otherwise} \\ \end{array}\right. } \end{aligned}$$

be the proportion of offspring that survive to adulthood. (None, if the population is already above carrying capacity. All, if the difference between the reproducing population size and the carrying capacity exceeds the new births. If the population is approaching carrying capacity, juvenile mortality scales proportionally so that the population will hit carrying capacity but not exceed it.)

Then

$$\begin{aligned}&S_1 + c \Delta S_1 \rightarrow S_1 \\&S_2 + c \Delta S_2 \rightarrow S_2 \\ \end{aligned}$$

We assume there is no vertical disease transmission, so all new hosts begin in the susceptible category. We assume that the host population is iteroparous, such that the new offspring and the existing adult population both carry over to the next generation. If the new population would exceed the carrying capacity, we assume the limited space or supplies reduces the number of successful offspring so that the population exactly reaches the carry capacity by reduction in juvenile survival rather than population-wide competition that could also reduce the adult population.

The carrying capacity is therefore what drives the interspecific host competition. Because births of both species are summed and then normalized by the total number of births, the higher the birthrate of one host, the larger a fraction of the available space it will capture during the breeding event. Similarly, the lower the death-rate of a host, the less space it frees up for the next breeding event. Even if one host species would be able to sustain a stable population on its own, the presence of a more fit competitor can lead to the extinction of the less fit type by driving its effective birth rate down.

### Immune-reproductive trade-offs and boundary conditions

We assume that host type 1 is evolutionarily stable in the absence of the disease; an uninfected monoculture population below the carrying capacity will have at least as many births as deaths each cycle. In a continuous version of this model where births and deaths happened simultaneously, this might be defined by $\alpha _{1-} \ge \mu _{1-}$ . However in our model, the population spends many days decreasing due to deaths before the next breeding event occurs. The population exponentially decays throughout the cycle, and then jumps up during the breeding event. The number of new host births is proportional to the number of hosts at the start of the breeding event, which will be the lowest value of any other time during the cycle. Thus, the birth rate needs to be high enough that the surviving hosts can compensate despite their diminished numbers. Taking this into account, we get the condition

$$\begin{aligned}&\alpha _{1-} \ge \frac{1-(1- \mu _{1-})^{t_c}}{(1-\mu _{1-})^{t_c}} \\ \end{aligned}$$

Which is a higher bound on $\alpha _{1-}$ than the simpler one above, but will be close to it if $\mu _{1-}$ and $t_c$ are small.

To implement the scenario in which type 2 has increased resistance and tolerance to the disease at the expense of overall fecundity, we implement the following boundary conditions:

$$\begin{aligned}&\beta _1> \beta _2 \\&0 = \gamma _1< \gamma _2 \\&\mu _{1-} = \mu _{2-}< \mu _{2+} < \mu _{1+} \\&\alpha _{1-}> \alpha _{2-}> \alpha _{2+} > \alpha _{1+} \end{aligned}$$

Type 2 hosts are less likely to contract the disease, and are able to recover from it, while type 1 lack the immunological strength to eradicate it completely. Additionally, while both types of host are weakened by the disease, type 2 suffer fewer negative effects. However, this stronger immune response comes at the cost of reducing their birth rate when compared to healthy type 1 hosts.

Due to the heterogeneous population, there is ambiguity in defining $R_0$ for the disease. The two types of host have different transmission rates and durations of infection, and will therefore be responsible for different amounts of disease spread. To resolve this, we define several related values. Let $R_0^j$ be the $R_0$ of the disease in a homogeneous population of type *j* hosts: the average number of hosts infected (indirectly, through vectors) from a single infected host in a population consisting entirely of type *j* hosts.

$$\begin{aligned}&R_0^1 = \frac{\delta _1 \beta _1 a b_1^2}{\mu _v \mu _{1+}} \\&R_0^2 = \frac{\delta _2 \beta _2 a b_2^2}{\mu _v (\mu _{2+}+\gamma _2)} \end{aligned}$$

We simplify the equation for $R_0^1$ since $\gamma _1 = 0$. We define *w* to be the frequency of host type 1: $w := (S_1 + I_1)/H$. Then $R_0$ for the vectors is

$$\begin{aligned} R_0^v = R_0^1 w + R_0^2 (1-w) \end{aligned}$$

which will also be the effective $R_0$ of the disease for the hosts in the mixed population.

For simplicity of results, we restrict to the case where type 1 is more infectious overall than type 2, in particular $R_0^1 > R_0^2$. This allows us to avoid edge cases in simulation outcomes which are beyond the scope of this paper. We intend to lift this restriction and study these outcomes in future work.

#### Note

Although usual epidemiological model formulations can rely on the value 1 as the boundary condition for $R_0$ to determine the epidemic potential of an outbreak, in this case we are calculating effective $R_0$ in a dynamic host population, such that the decrease in disease spread due to saturation from recovered hosts and already infected hosts increases the actual thresholds. More accurate criteria require a technical and somewhat cumbersome analysis, which we leave for a future paper.

## Results

The long-term behavior of the model is sensitive to parameter values, but does not depend on the initial conditions, provided the starting size for each population is nonzero. Thus, we focus on presenting analysis of the parameter space in the competition between hosts, rather than sensitivity to initial conditions.

We classify outcomes for the system into one of four categories:

1. *Failure to establish* The invading host 2 population asymptotically goes to zero, while the host 1 population remains near the carrying capacity.
2. *Coexistence* Both host types survive at a stable level without going extinct.
3. *Competitive exclusion* The host 1 population decreases asymptotically to zero and is replaced completely by type 2 hosts.
4. *Extinction* Introduction of infection alters the system such that both host populations asymptotically go to zero.

We define a set of parameters that lead to coexistence, which we refer to as the ‘default parameters’, shown on Table 4. All figures and numerical results are made using the default values for each parameter except when otherwise specified.

**Table 4:** Default Parameters.

Transmission	Host 1		Host 2		Vector
	$\beta _1 = 0.008$		$\beta _2 = 0.005$		$\delta _j = 0.05$
Recovery	$\gamma _1 = 0$		$\gamma _2 = 0.003$		
	Uninfected	Infected	Uninfected	Infected	
Death	$\mu _{1-}=0.001$	$\mu _{1+} = 0.0025$	$\mu _{2-} = 0.001$	$\mu _{2+} = 0.0011$	$\mu _v = 0.02$
Birth	$\alpha _{1-}=0.002$	$\alpha _{1+}=0.0003$	$\alpha _{2-}=0.0018$	$\alpha _{2+}=0.0014$	$\alpha _v = 0.02$

Additionally, we set the carrying capacity $\kappa = 15000$, days per year $t_c = 365$, bite rate $b_j = 1$, and as initial conditions set $S_1 = 14000, S_2 = 1300, I_2 = 200, S_v = 14000$, and all other initial populations to 0. Although in general the vector transmission rate from the host types, $\delta _1$ and $\delta _2$, need not be equal, for simplicity here we set them both equal to 0.05.

**Figure 1:**
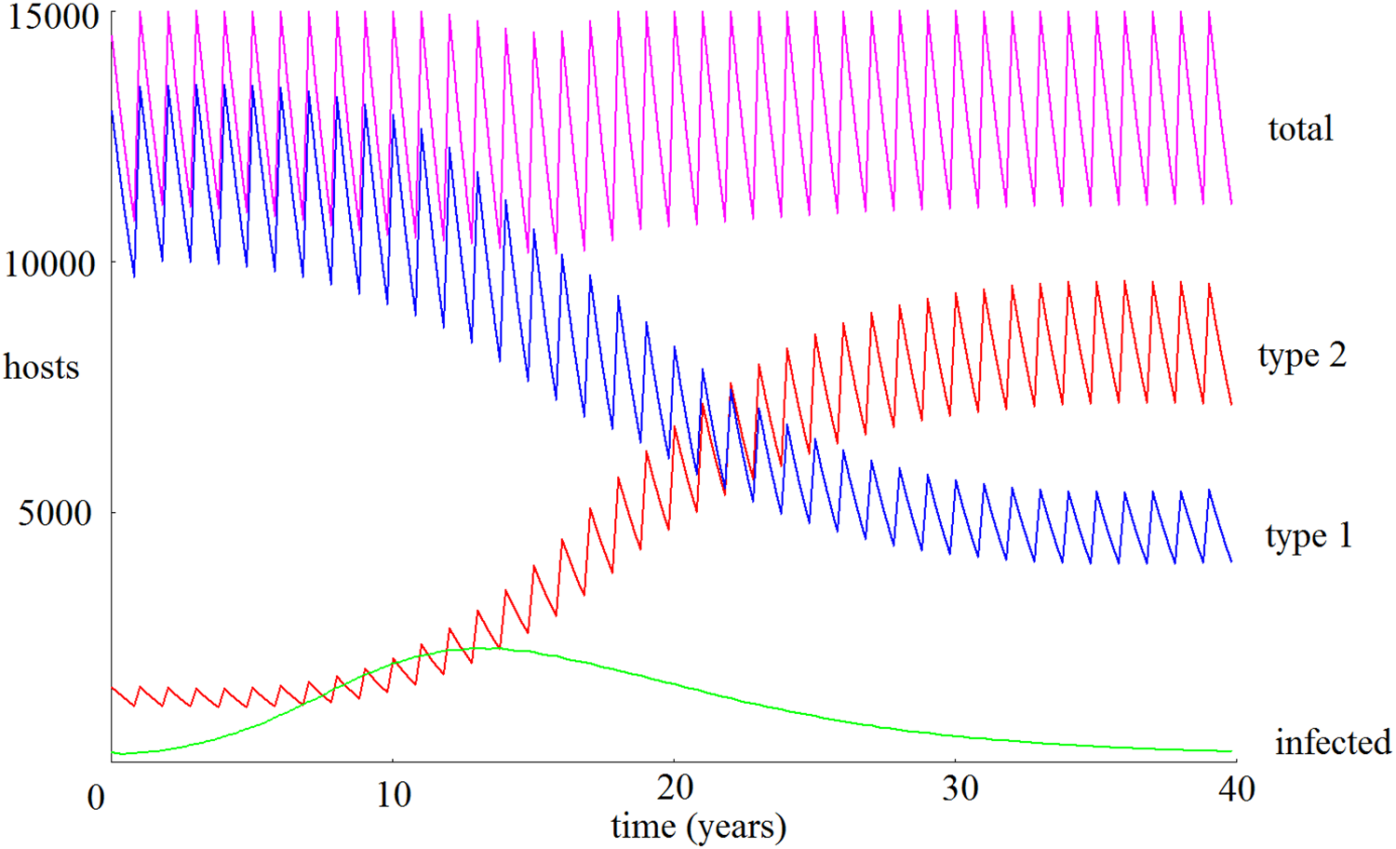
A precise rendering of the host populations over 40 years using default parameters. Default parameters are selected to provide conditions of Coexistence between the two host populations, as seen here (blue and red curves). The host population curves are seen to zigzag due to the annual breeding cycle. Under this scenario, disease prevalence (green curve) decreases as the more robust type 2 host population increases.

As intended, our default parameters yield Coexistence between the two host types (Fig. 1).

**Figure 2:**
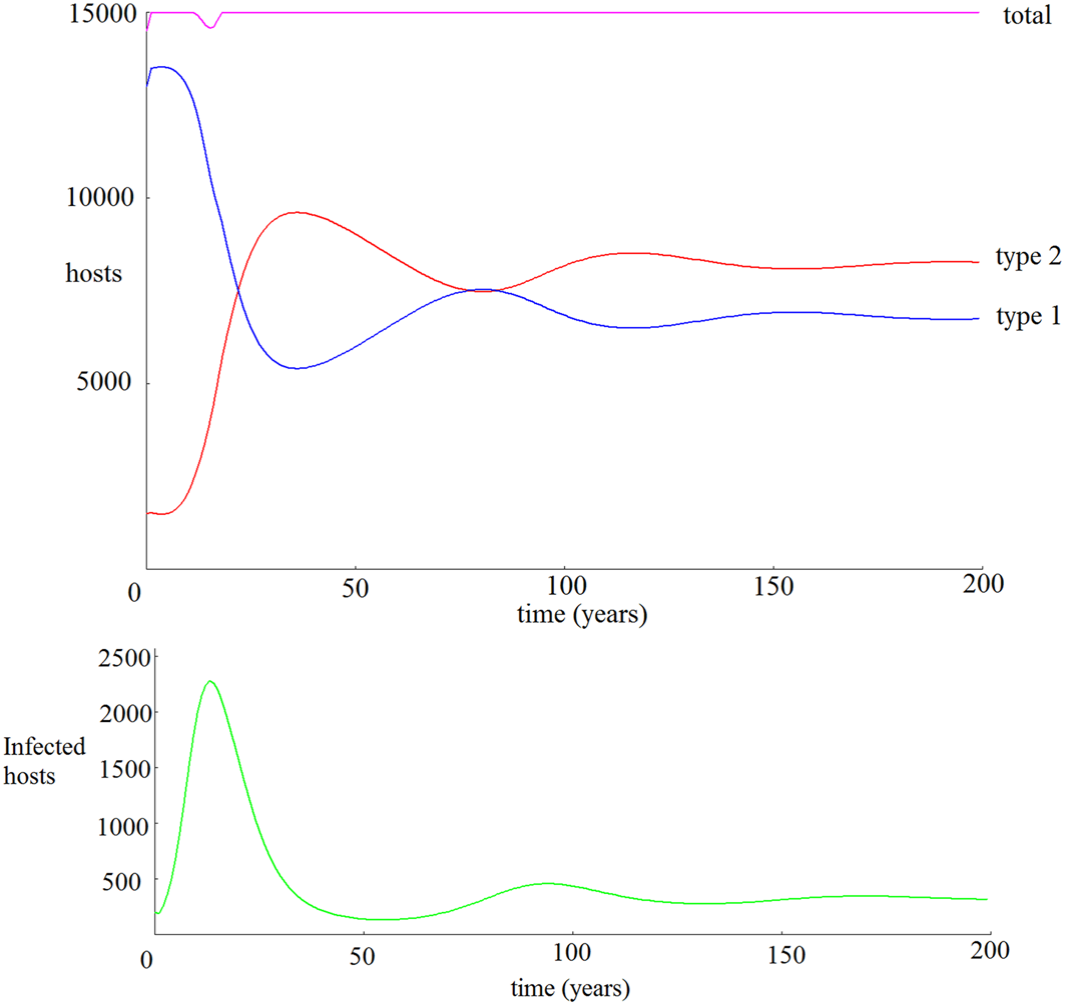
A smoothed, longer-term projection of the host populations (blue and red curves) over 200 years using default parameters. Under this longer time frame, we observe damped oscillations in the infection prevalence (green curve) before the populations stabilize to Coexistence.

To observe the longer-term trends, we use the same default parameters and sample data points once each year immediately after the breeding event (Fig. 2), thereby smoothing out the yearly cycles in the population. Under this default scenario, the initial infection grows into an epidemic which reduces the host 1 population, which then causes the outbreak to recede. This in turn allows the host 1 population to recover until it triggers another smaller epidemic, again reducing their population. These oscillations gradually decrease in magnitude and the population approaches a stable equilibrium (we leave analytic characterization of these dynamics to future work). Because the total host population reaches the carrying capacity after each breeding event, the host 2 population varies inversely with the host 1 population.

**Figure 3:**
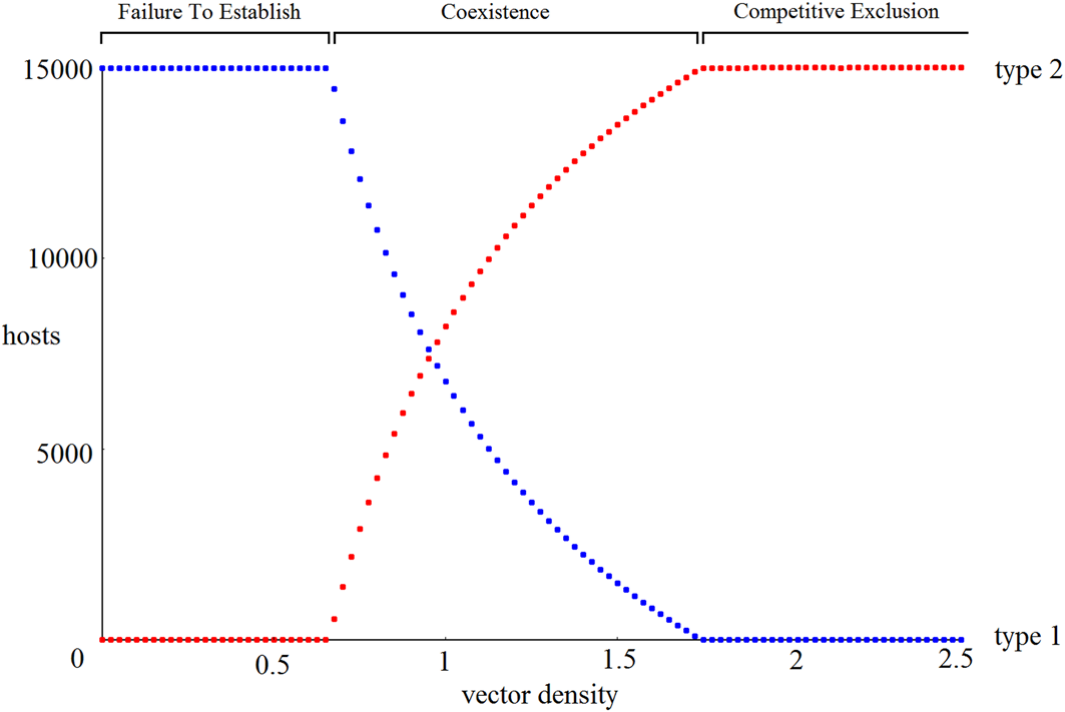
Hosts populations after 200 years as a function of vector density. Due to parallel action in the system dynamics of several variables in driving the force of infection, a nearly identical result would occur if the x axis instead presented a fixed ratio for any of the following pairs of parameters: $\{\beta _1, \beta _2\}, \{\delta _1, \delta _2\},$ or $\{b_1^2, b_2^2\}$.

Similar behavior is observed over a wide range of parameters, with the equilibrium frequency of host 1 depending primarily on parameters that influence the spread of infection. Figure 3 shows the 200 year projected results for simulations using default parameters for every parameter except $\alpha _v$, which we set equal to 0.2a, where a is the desired vector density.

We observe that the host outcome is strongly dependent on vector density. Low vector density leads to the Failure to Establish outcome. As vector density increases, Coexistence occurs, with the frequency of each host population changing continuously with vector density. For high density, we observe the Competitive Exclusion outcome.

**Figure 4:**
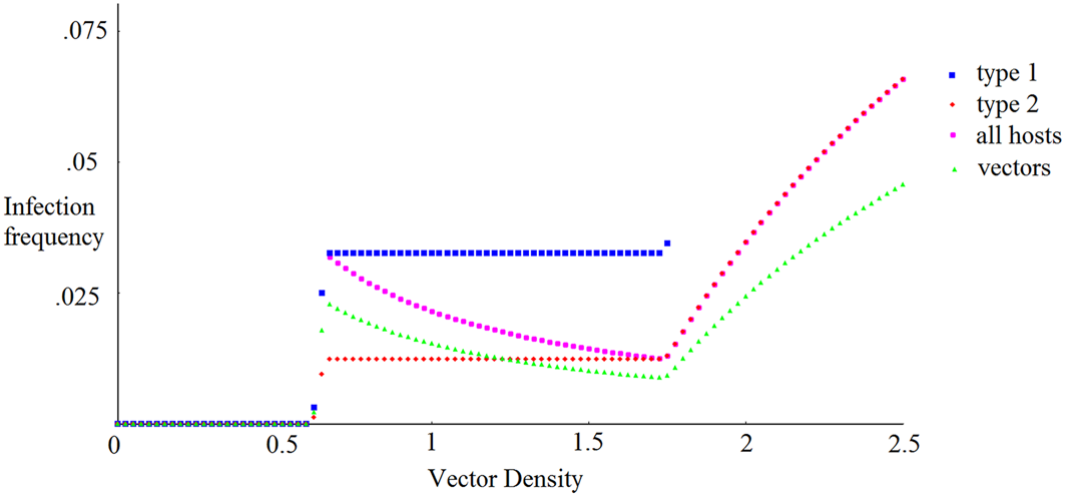
Infection frequency as a function of vector density, shown for vectors, each host type, and both host types together, measured after 200 years.

These outcomes are rooted in the infection dynamics. Figure 4 shows infection frequencies as a function of vector density. When the vector density is low, the pathogen is unsuccessful at spreading. At the same threshold observed in Fig. 3, there is a discontinuous jump in infection success. Afterwards, contrary to expectation, the infection rates for vectors and all hosts actually decreases as vector density increases. This decrease can be attributed to something akin to Simpson’s paradox: each species of host maintains a constant level of infection in this region (Fig. 4; we leave discussion of the technical details causing this constant level for a future work). Because type 1 hosts are more likely to be infected than type 2, the average infection level of the whole population increases or decreases alongside the type 1 frequency. Since vectors interact with hosts proportionally to their frequency in the host population, this in turn causes the vector infection rate to decrease as well. Once the type 1 hosts go extinct, the rate of infection in the host 2 population increases with vector density, as we would normally expect.

Extinction never occurs under the range of parameters shown in these figures, since even if every host becomes infected, the host 2 population can replace itself faster than it dies. Extinction can occur under different birth or death rates that do not guarantee demographic replacement for host 2. When populations are below the carrying capacity, the birth and death equations are proportional to the current population size, so the host populations will grow approximately exponentially, assuming the infection level is stable. If the exponent is positive, the population will increase until it reaches the carrying capacity. If the exponent is negative, the population will asymptotically approach 0. An approximation for this exponent would be the average birth rate of the host type, given the average frequency of infected and uninfected individuals minus the average death rate. (Note: While this is a reasonable approximation for most parameters, it is not quite accurate since breeding happens after a year of cumulative host death, therefore the size of the population that reproduces is smaller than its average size throughout the year.)

**Figure 5:**
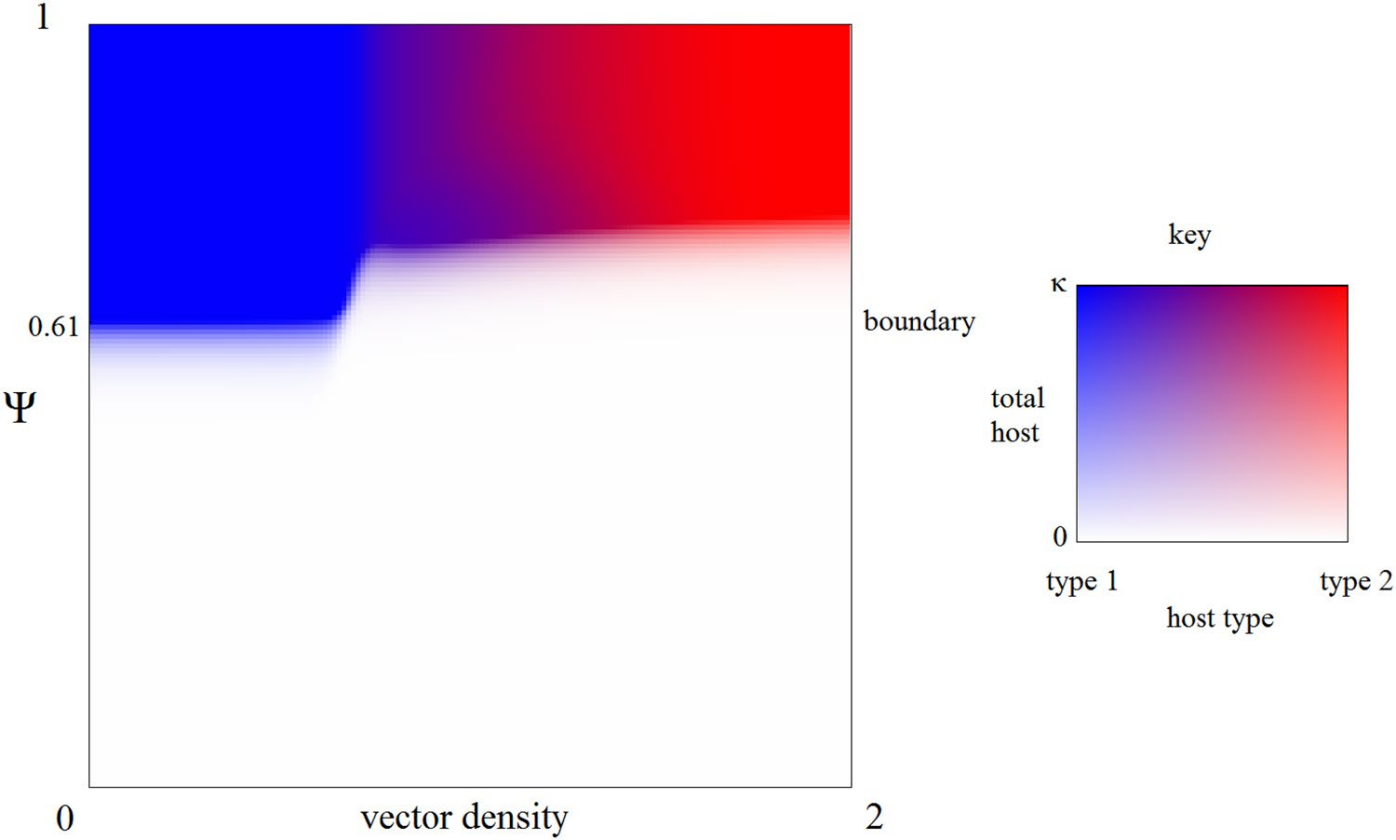
The representation of the two types of hosts after 200 years as the factor by which we multiply the intrinsic birth rate of both hosts, $\Psi$, and vector density vary.

In order to better show how the epidemic interacts with the host frequency equilibrium and extinction, we allow the birth rates of both host types to vary. In particular, we multiply the default values for $\alpha _1$ and $\alpha _2$ by the same fixed value $\Psi$, and construct a plot that shows the population state for a simulation after 200 years, as $\Psi$ and vector density vary (Fig. 5). For generality, we show outcomes where $\Psi$ varies from 0 to 1, although only values above 0.61 satisfy the boundary condition for uninfected host 1 being evolutionarily stable.

All four possible outcomes are achievable through different combinations of the $\Psi$ and vector density (Fig. 5). When birth rates are low, Extinction can occur, regardless of vector density, though the threshold for survival is seen to be vector density dependent. When birth rates are high and vector density is low, no epidemic occurs and we see Failure to Establish. Under intermediate values of vector density, we see Coexistence between both host types. Additionally, we observe a continuous gradient of host frequencies, with more type 2 hosts as vector density increases. When density is high, we see Competitive Exclusion, with only type 2 hosts surviving.

We also observe a phase transition between extinction and reaching carrying capacity as the birth rate varies, with a small transition region between them. This occurs since populations near the phase transition will exponentially grow or decay with an exponent very close to 0, so the time required to reach equilibrium will exceed the 200 year horizon presented in Fig. 5. As the time horizon increases, the boundary between the extinction (white) and non-extinction (colored) regions in the figure becomes increasingly sharp (not shown).

## Discussion

As an increasing number of animal (and plant) species move (or are transported) around the world, the dynamics of biological invasions get more complicated^39^. When those invasions are further complicated by involving pathogens or parasites, they can reshape the nature of entire ecosystems^40,41^. Our model has demonstrated that invasive hosts carrying vectorborne pathogenic “wingmen” can drastically alter the dynamics of invasions, meaningfully shifting the likely outcomes among the options: Failure to Establish, Coexistence, Competitive Exclusion, and Extinction. Although failed invasions are difficult to study in detail, and may go completely unnoticed due to their lack of substantial impact, examples of successful invasions assisted by a disease have been observed in several cases such as in squirrels^27,28^, moose and deer^30^, and crayfish^31^, among others^23^. The study of human infectious diseases further demonstrates how the global spread of vectorborne pathogens may easily be driven by the mobility of infected hosts, rather than solely by the expansion of habitat range of vectors (e.g. the global spread of Zika virus in 2016 as infected human hosts traveled around the world to places where competent vectors already existed, but no virus had yet been introduced^42^.)

Existing studies have already considered the opposite scenario from the one here presented^43,44^ in which native hosts have increased resistance to infection relative to their more susceptible would-be invaders. Invasion success in this case is unlikely, even if the invading hosts have a competitive fitness advantage in the absence of infection^43^. In that scenario, rather than acting as a wingman to the invaders, the pathogen acts as a protective barrier against invasion. Although their model is different from ours in many ways, (e.g. studying a direct transmission, SI disease model using a stochastic cellular automaton), our conclusions are mutually consistent. The success of either the native or invading host are both possible, as is stable coexistence, but that the outcome depends on the relative demographic and etiological factors in each host type, where increasing pathogen transmissibility shifts selective pressures and competitive advantage to favor the disease-resistant host.

Many models of vector-borne disease spread have also considered a dilution effect, where an increase in host diversity decreases the spread of infection borne by generalist vectors, typically by decreasing the density of highly competent hosts for the disease and mixing them with less competent hosts^45–48^. While carrying a native pathogen into a habitat with an additional novel host does increase available host diversity, dilution would only occur in the case in which the native host is less susceptible to infection than the invaders. Increased native susceptibility would lead the native host to amplify, rather than dilute, the disease risks to both populations (as our results show; Fig. 5). Of course, this can be further complicated by factors such as vector feeding preferences^49,50^. If vectors focus more attention on a single host type, vector bites will be more concentrated on a small group of hosts, increasing the contact rate between infected hosts and uninfected vectors. Thus, the dilution effect of adding more host types to an ecosystem may be overestimated by models that do not consider feeding preferences in generalist vectors.

Our model contains terms for vector bite rate $b_j$, which are analogous to vector feeding preference but fail to conserve total number of bites per vector as the host frequencies change. In this way, our model highlights the need to consider the full ecological, evolutionary, and epidemiological complexity of systems in being able to understand and predict the interactions among, and trajectories of, host populations.

In our model, when disease outbreaks are similarly likely in both host populations (i.e. when large outbreaks occur in both host types or else in neither), only one host type should ultimately persist. Using vector density as the dial by which to tune the relative force of infection, we see that when the introduced pathogen is unlikely to spread in either host type, type 1 hosts will outcompete type 2. Conversely, when the infection is likely to spread in both host types, type 2 hosts dominate. Of course, vector density yields these observed results due to its action on the force of infection in each host population, but other factors in the model similarly impact the force of infection. Therefore, tuning any of these factors ($\delta _j,\beta _j,b_j$) would result in similar system-wide dynamics.

The evolutionary dynamics, in fact, depend very little on the actual disease severity, except in so far as severity affects the force of infection. For example, in any modeled scenario, if we were to multiply both the type 1 death rate attributable to infection, $\mu _{1+}$, and the type 1 infection rate, $\beta _1$, by a factor of 100 (thereby keeping $R_0^1$ constant), there would be no change in the predicted outcome (excepting edge cases). If the disease fails to spread, relative death rate is irrelevant to the evolutionary outcome. If the disease spreads among the host 2 population then the host 1 population will still die out and the higher death rate will simply hasten this inevitable outcome. Even in the Coexistence outcome, the equilibrium frequency of type 1 hosts won’t change significantly; a more deadly disease will lower the equilibrium infection level required to keep the host 1 population in check, but not the resulting frequency of type 1 hosts. (Future work is underway to explore the analytic boundary conditions of these dynamics.)

An important result from our model is that increasing transmissibility of the infection increases the relative fitness of type 2 hosts, and therefore actually increases their equilibrium frequency (assuming extinction does not occur). The competitive evolutionary benefit outweighs the epidemiological cost. This wingman pathogen dynamic can therefore play a pivotal role in determining whether invasion leads to Coexistence or Competitive Exclusion. The more easily the disease spreads, the higher the frequency of the invasive host species in the resulting equilibrium compared to the native host, and this change in frequencies happens in a continuous way. The distinction between survival and extinction, however, depends more on the birth and death parameters, and happens in a discontinuous way: the population as a whole either goes to the carrying capacity, or to extinction, with no equilibrium in between. We attribute this to our choice for the vector/host interaction rate to depend on the ratio of vectors to hosts. If our model had instead assumed that decreasing host population necessarily implies decreasing host density, then resulting in decreasing opportunities for transmission, this would slow the spread of disease and should lead to the persistence of small host populations in cases where our model leads to extinction.

## Conclusion

While invasion success is determined by a complicated and diverse set of environmental and ecological factors, pathogens carried by invasive hosts can alter the competitive landscape and significantly alter their probability of establishment. This is especially true in cases where, either due to accident or coevolutionary selective pressures, the pathogens cause only minor fitness costs in the invaders, but cause substantial costs to native hosts. We have shown how some cases of such vectorborne “wingman” pathogens allow for stable Coexistence of both host types where, in their absence, the invading species would have simply failed to establish a persisting community, and can even shift the balance entirely allowing for the displacement of the native entirely (Competitive Exclusion) instead of failing to establish in their new habitat. These results clearly demonstrate the need for more nuanced, community ecology perspectives on the epidemiological-ecological dynamics of invasions in a global world of increasing species movement of hosts, vectors, and pathogens.

## Data Availability

All simulations and figures were generated by original code by the authors, available at https://github.com/kazarraha/SIRVectorModel.
